# Assessment of local supply chains and stock management practices for trauma care resources in Ghana: a comparative small sample cross-sectional study

**DOI:** 10.1186/s12913-021-06063-6

**Published:** 2021-01-13

**Authors:** Godfred Boakye, Adam Gyedu, Melissa Stewart, Peter Donkor, Charles Mock, Barclay Stewart

**Affiliations:** 1 Department of Surgery, School of Medicine and Dentistry, Kwame Nkrumah University of Science and Technology, Kumasi, Ghana; 2 Ghana Armed Forces, Accra, Ghana; 3 University Hospital, Kwame Nkrumah University of Science and Technology, Kumasi, Ghana; 4 Foster School of Business, University of Washington, Seattle, USA; 5 Harborview Injury Prevention & Research Center, Seattle, WA USA; 6 Department of Surgery, University of Washington, Seattle, WA USA; 7 Department of Global Health, University of Washington, Seattle, WA USA; 8 Department of Surgery, Division of Trauma, Burn and Critical Care Surgery, UW Medicine, Harborview Medical Center 325 9th Ave, Box 359796, Seattle, WA 98104 USA

**Keywords:** Supply chain, Operations, Stock management, Trauma, Surgery, Ghana

## Abstract

**Background:**

Injuries are a major public health problem globally. With sound planning and organization, essential trauma care can be reliably provided with relatively low-cost equipment and supplies. However, availability of these resources requires an effective and efficient supply chain and good stock management practices. Therefore, this study aimed to assess trauma care resource-related supply management structures and processes at health facilities in Ghana. By doing so, the findings may allow us to identify specific structures and processes that could be improved to facilitate higher quality and more timely care.

**Methods:**

Ten hospitals were purposively selected using results from a previously performed national trauma care capacity assessment of hospitals of all levels in Ghana. Five hospitals with low resource availability and 5 hospitals with high resource availability were assessed using the United States Agency for International Development (USAID) Logistics Indicators Assessment Tool and stock ledger review. Data were described and stock management practices were correlated with resource availability.

**Results:**

There were differences in stock management practices between low and high resource availability hospitals, including frequency of reporting and audit, number of stock-outs on day of assessment (median 9 vs 2 stock-outs, range 3–57 vs 0–9 stock-outs, respectively; *p* = 0.05), duration of stock-outs (median 171 vs 8 days, range 51–1268 vs 0–182 days, respectively; *p* = 0.02), and fewer of up-to-date stock cards (24 vs 31 up-to-date stock cards, respectively; *p* = 0.07). Stock-outs were common even among low-cost, essential resources (e.g., nasal cannulas and oxygen masks, endotracheal tubes, syringes, sutures, sterile gloves). Increased adherence to stock management guidelines and higher percentage of up-to-date stock cards were correlated with higher trauma resource availability scores. However, the variance in trauma resource availability scores was poorly explained by these individual factors or when analyzed in a multivariate regression model (r^2^ = 0.72; *p* value for each covariate between 0.17–0.34).

**Conclusions:**

Good supply chain and stock management practices are correlated with high trauma care resource availability. The findings from this study demonstrate several opportunities to improve stock management practices, particularly at low resource availability hospitals.

## Background

Injuries are a major public health problem globally, incurring more than 11% of all disability-adjusted life years incurred annually [1, 2]. Nearly 90% of this burden falls on low- and middle-income countries (LMICs), which are least prepared to provide trauma care [3]. Opportunely, with sound planning and organization, effective trauma care can be reliably provided with relatively low-cost equipment and supplies [4–6].

However, availability of these equipment and supplies requires an effective and efficient supply chain [7–9]. Supply chains include the structures and processes for sourcing of equipment and consumables, purchasing and procurement, transport, and distribution of products [10, 11]. The ways in which these structures and processes interact with one another and are organized significantly impacts the availability of essential trauma care resources for patients when needed [8, 12]. Examples of systematic trauma care supply chain assessments and targeted improvements in LMICs have not been published. However, other essential services routinely perform and improve their supply chains to strengthen reliable service delivery [13]. As example, family planning resource-related supply chain assessment and improvement interventions in Rwanda reduced the stock-outs of contraceptives through best practice implementation orchestrated by the government and other key partners [14]. Thus, it might be possible to do similar work for essential trauma care in an effort to reduce stock-outs of resources required to provide life- and limb-saving care. Although no essential trauma care resources require unique supply chain management practices, trauma care resources have not been prioritized by public health agencies, governments or healthcare systems like other global health priorities (e.g., neglected tropical diseases; maternal neonatal and child health) [15, 16]. As a result, there may be supply chain vulnerabilities and stock management practices that impact the delivery of care to the injured and require attention. Further, given the similarities between essential trauma care resources and other health products, there is great potential for important and positive knock-on effects of improving trauma care-related supply chain management practices on other priority health conditions (e.g., resuscitation equipment and supplies for sepsis, surgical services for maternal care).

Multiple supply chain and stock management structures have been developed by healthcare systems in LMICs. However, these structures have commonly been designed to fit the administrative structures of the healthcare system instead of key technical or operational considerations that impact the supply chain [17]. As a result, the processes deployed to forecast demand, manage warehouse or medical stores flow, rotate inventory, perform cycle counts and audit stock management practices are inefficient and not tailored to the unique requirements of specific resources or product utilization. These inefficiencies further exacerbate well-documented challenges in healthcare supply chain management in LMICs, including diffuse accountability, uncertainties in financing, unnecessarily complex workarounds, long resupply intervals, inadequate funding of operating costs, and insufficient incentivization schemes for supply chain management personnel [18–20]. In response, several opportunities to improve supply chain management structures have been proposed: minimize tiers within the system, increase the frequency of replenishment to avoid maintenance of large stocks, streamlining information flows, ensure transparent governance, and perform system audits [13, 14, 17, 18, 21].

A nationwide assessment of trauma care capacity was performed in Ghana in 2015 [8, 12]. The study also undertook a root cause analysis to identify the factors that contributed to resource non-availability. The study reported that a lack of supply chain management structures and processes was often responsible for stock-outs of non-drug consumables (e.g. airway supplies, chest tubes, laboratory reagents) [8, 12, 22]. However, that data gathered did not allow specific recommendations to be made regarding ways to improve supply chain management practices and no dedicated stock management sytem performance audit was undertaken.

To address this gap and comply with recommendations to perform system audits, this study aimed to assess trauma care resource-related supply management structures and processes at health facilities in Ghana. Specifically, the study aimed to measure the stock of essential trauma care supplies at the day of survey and in the 6 months prior, measure key stock management performance indicators from stakeholder interviews and by observation and measure the correlation between select performance indicators and stock. By doing so, the findings may allow us to identify specific structures and processes that could be improved to facilitate higher quality and more timely care.

## Methods

### Setting

Ghana has 16 regions divided into 257 districts. Most districts have several primary health centers (PHC) and a government or mission hospital that serves as a district (first-level) hospital. PHCs provide only basic public health and primary care services; therefore, they were not included in the study. District-level hospitals are staffed by medical officers and nurse anesthetists, usually offer surgical services and have between 50 and 100 beds. Injuries requiring more complex care are referred to one of the regional or five teaching hospitals. In addition to medical officers and nurse anesthetists, regional hospitals are staffed by specialist providers (general and orthopedic surgeons) and contain between 100 and 400 beds. Surgical services offered at regional hospitals are broader in scope. There are five tertiary care hospitals in Ghana (one of which doubles as a regional hospital); all are affiliated with a medical school or residency program and offer more specialized care.

The public procurement law of Ghana, Act 663 of 2003, as amended, Act 914 of 2016, provides the framework for the health supply chain. Operations experts have noted that this document has inadequate regulations and framework for procurement, transportation, storage and distribution, disposal and reverse logistics of health commodities [23]. Regardless, the law directs health commodities to be managed by a three-tier system: Central Medical Store, regional medical stores, and service delivery points [9]. Ministry of Health coordinates procurement and the Ghana Health Service oversees the operations of the supply chain [24]. These activities are linked to the hospitals through a combined public and contracted transportation system. The Central Medical Store is managed by the Ministry of Health. Each regional medical store is managed by the respective Regional Health Administration [25]. In exceptional cases and only after applying to the Ministry of Health, tertiary hospitals and regional hospitals are allowed to design, organize and execute their own supply chains and work independently with private vendors. They may also procure their commodities directly from the Central Medical Store.

### Sample strategy

Ten hospitals were purposively selected using results from a previously performed national trauma care capacity assessment of hospitals of all levels in Ghana [8]. In that assessment, the availability of trauma care resources within 40 hospitals were rated by local stakeholders and direct observation. The assessment determined that ineffective supply chain management practices significantly contributed to a lack of resource availability to injured adults and children when needed [8, 12]. To identify both effective and ineffective supply chain management practices, we selected five hospitals with low resource availability scores for 51 trauma care resources and five hospitals with high resource availability scores for trauma care resources. Trauma care resource availability scores used for this study were the directly derived from the rating scheme of the previous assessment: 0 – absent; 1 - inadequate, available to less than half of those who need it; 2 – partially adequate, available to more than half, but not to most who need it; 3 – adequate, present and readily available to almost everyone in need and used when needed [8]. Low resource availability was defined as being in the lowest quartile for total trauma care resource availability score (i.e., sum of 51 resource availability scores) and high resource availability score was defined as being in the highest quartile for total resource availability score.

We stratified the 40 hospitals based on their level of care (i.e., tertiary, regional, district/first-level hospital) and their resource availability scores. The 10 hospitals were then sampled at random, assuming they met the following criteria: three district hospitals with low resource availability scores; three district hospitals with high resource availability scores; one regional hospitals with a low resource availability score; one regional hospital with a high resource availability score; one tertiary hospital with low resource availability score; and one tertiary hospital a high resource availability score.

At each of the sampled hospitals, our objectives were to measure the stock of essential trauma care supplies at the day of survey and in the 6 months prior, measure key stock management performance indicators from stakeholder interviews and by observation and measure the correlation between select performance indicators and stock.

### Assessment tools and data collection

Data on supply chain structure and processes of the selected hospitals were collected by using a structured questionnaire adapted from United States Agency for International Development (USAID) Logistics Indicators Assessment Tool (LIAT) [26]. The USAID LIAT was selected as the assessment tool given its widespread use for healthcare supply chain assessment in LMICs and prior use in Ghana to study stockouts of essential medicines [27]. We modified the LIAT for the Ghanaian first-level hospital context and reworded some of the prompts to be specific for the trauma care resources described by the previous nationwide trauma care capacity assessment [8]. The LIAT was used to assess the structure and processes of local supply chains at the selected hospitals through face-to-face key informant interviews with all hospital personnel who engage with the local supply chain to minimize the risk of reporting bias by triangulating responses. The LIAT contains 46 questions subdivided into three domains. The domains consist of: i) general stock management (e.g., use of stock system, information technology usage, stock management training, frequency of stock accounting and supervision), supply chain performance (e.g., stock-out rates, duration of stock-out); and iii) storage structures and delivery processes. We compared responses to the LIAT with stock ledgers to further reduce the risk of reporting bias. Six months of stock ledgers were reviewed for number and duration of stock-outs for each trauma care resource. No identifying information was recorded.

### Data analysis

Data collected were on paper forms and doubly transcribed to Microsoft Excel v16 (Microsoft Corporation, USA). Differences were rectified using the original form. Supply chain management practices of both groups (five hospitals with resource availability for non-drug consumables and five hospitals with high resource availability scores for non-drug consumables) were described using Stata v14 (StataCorp, USA). LIAT scores from each hospital group were compared and specific successes and challenges were highlighted. The relationship between resource availability and stock management practices was assessed with Chi square test and linear regression. Differences between hospitals were not tested given the purposively small sample.

### Ethical considerations

Approval for the study was provided by the ethical committee of the Kwame Nkrumah University of Science and Technology and the University of Washington Institutional Review Board. All data were anonymously recorded. Permission was granted from each Regional Health Directorate and individual hospital administrators (medical superintendents or administrators) before hospital visits.

## Results

### General stock management

Nearly all hospitals had a stock management record system (e.g., stock card and ledger) that included information regarding stock on hand, quantities used per unit time, losses, and anticipated adjustments (Table 1). Most hospitals verified the ledger and reported the results quarterly. There were minimal differences between low and high resource availability hospitals. Low resource availability hospitals more often had dedicated supply chain management workshops and on-the-job training structures for staff than high resource availability hospitals, which reported less on-the-job training. There were also little differences in the frequency of emergency orders between the hospital groups. Almost all hospitals had flexible and current need responsive methods for determining re-supply quantities and used a combination of local contractors and facility staff for product delivery. Hospitals in both groups most commonly required 2–4 weeks lead time for ordering, procurement and delivery. The frequency of supervision visitation was not consistent across hospitals and was less frequent in the low resource availability hospital group.

**Table 1:** General supply chain management structures and processes at low and high resource availability hospitals in Ghana

	Low resource availability hospitals		High resource availability hospitals	
	n	(%)	n	(%)
**Stock keeping forms**				
Stock card	5	(100)	5	(100)
Stock ledger	5	(100)	5	(100)
**Logistics management information system (LMIS)**				
LMIS system present and maintained	2	(40)	3	(60)
**LMIS fields used**				
Stock on hand	5	(100)	4	(80)
Quantities used	5	(100)	5	(100)
Losses and adjustments	5	(100)	5	(100)
**Frequency of LMIS reporting**				
Quartely	5	(100)	4	(80)
Semi-annually	0	(0)	1	(20)
**Most recent LMIS report submitted**				
Within a month	1	(20)	0	(0)
2 months ago	4	(80)	4	(80)
3 months ago or more	0	(0)	1	(20)
**Stock management training**				
During logistics workshop only	2	(40)	4	(80)
On-the-job training only	0	(0)	0	(0)
Both	3	(60)	1	(20)
**Frequency of emergency orders in last 3 months**				
Less than 3 orders	3	(60)	2	(40)
3 or more orders	2	(40)	3	(60)
**Determination of re-supply quantities**				
Set formula	1	(20)	1	(20)
Flexible and current need responsive method	4	(80)	4	(80)
**Party responsible for resource delivery**				
Local contractor	4	(80)	5	(100)
Government transportors	2	(40)	2	(40)
Facility staff	4	(80)	5	(100)
**Lead time for resources**				
Less than 2 weeks	1	(20)	2	(40)
2 weeks to 1 month	3	(60)	3	(60)
More than 1 month	1	(20)	0	(0)
**Most recent inspection by supervisor**				
Within the last month	1	(20)	2	(40)
1–3 months ago	2	(40)	1	(20)
3–6 months ago	1	(20)	2	(40)
More than 6 months ago	1	(20)	0	(0)

Table 2 demonstrates the prevalence of stock management system, including the presence of any and up-to-date stock cards. In general, stock cards were present for most resources. However, low resource availability hospitals lacked up-to-date stock cards compared to high resource availability hospitals. Further, day of assessment stock-outs were markedly less common in high resource availability hospitals.

**Table 2:** Trauma resource availability and stock management at low and high resource availability hospitals in Ghana

	Low resource availability hospitals			High resource availability hospitals		
Item	Number of facilities who had stock card for product	Number of facilities with stock card updated	Number of facilities experiencing stock out on day of visit	Number of facilities who had stock card for product	Number of facilities with stock card updated	Number of facilities experiencing stock out on day of visit
Airway (A) resources						
Oral airways	3	2	0	3	1	0
Nasal airways	3	1	1	2	0	0
Nasal cannula	5	3	1	5	2	0
Oxygen masks	5	2	2	4	2	0
Endotracheal tubes	5	2	3	4	2	0
Bougie	3	0	2	3	2	1
Endotracheal and tracheostomy ties	5	3	0	4	3	0
Yankauer tips	4	2	2	3	2	0
Suction tubing	4	2	2	3	2	0
Nasogastric tubes	4	2	1	4	2	0
Cervical collars	4	1	3	4	3	1
Breathing (B) resources						
Chest tubes	2	2	0	5	3	1
Underwater seal containters				4	2	0
Bag-valve-mask	4	2	1	5	3	0
Ventilator circuit tubing	1	1	0	2	1	0
Circulation (C) resources						
Blood pressure cuffs	5	3	0	3	2	1
Spirit (isopropyl alcohol)	5	3	0	4	2	0
Absorbent cotton wool	5	3	0	4	3	0
IV cannulas	5	3	0	5	3	0
IV fluid administration sets	4	2	0	5	3	0
Blood administration sets	3	2	0	5	3	0
Intraosseous needles				3	2	3
Central venous access sets	2	1	1	3	2	0
Syringes	5	2	0	4	3	0
Sharps disposable container	4	2	1	5	2	0
Cardiac monitor leads	2	0	1	2	1	0
Electrocardiogram leads	5	1	1	4	2	1
Electrocardiogram paper	1	1	1	3	2	0
Defibrillator pads	1	1	0	2	1	0
Urine dipsticks	5	3	0	3	2	0
Urinary catheters	5	3	0	5	3	0
Urinary and drainage catch-bags	5	3	0	5	3	0
Disability (D) and exposure (E) resources						
Splint padding material	2	2	0	3	2	0
Plaster of Paris	3	2	0	4	3	0
Gloves (examination)	5	3	0	5	4	0
Face masks	5	3	0	5	3	0
Eye protection	5	3	0	4	2	0
Adjunct and other resources						
Xray development supplies	1	1	0	4	2	0
Ultrasound gel	5	3	1	5	3	0
Iodine or chlorhexidine	1	1	0	3	2	0
Disposable needles	5	2	0	4	3	0
Otoscope tips	3	0	1	3	2	1
Glucose test strips	5	3	0	4	2	0
Finger stick lancets	5	3	1	5	3	0
Insulin needles	5	3	1	4	2	0
Surgical resources						
Scalpel blades	5	4	0	5	3	0
Surgical drains	2	2	0	4	3	1
Sutures	5	2	0	5	3	0
Sterile gauze	5	3	0	4	3	0
Sterile bandages	5	2	0	4	3	0
Gloves (sterile)	5	3	0	5	3	1

### Stock management performance

The high resource availability hospital group managed a median of 47 of the 51 trauma care resources assessed (range 35–51 resources) compared to 40 resources at low resource availability hospitals (range 34–51; *p* = 0.09) (Table 3). High resource availability hospitals more often had stock cards and up-to-date stock cards compared to low resource hospitals (median 44 vs 35 available stock cards and 31 vs 24 up-to-date stock cards, respectively). High resource availability hospitals were less likely to have a trauma care resource stock-out on the day of assessment (median 1 stock-out, range 0–5) than low resource availability hospitals (median 4 stock-outs, range 2–14; *p* = 0.05). Similarly, high resource availability hospitals had fewer stock-outs within the prior 6 months (median 2 vs 9 stock-outs, range 0–9 vs 3–57 stock-outs, respectively) and a few total number of days of trauma resource stock-outs (median 8 vs 171, range 0–182 vs 51–1268, respectively).

**Table 3:** Stock management and supply chain performance for trauma resources at low and high resource availability hospitals in Ghana

	Low resource availability hospitals		High resource availability hospitals		
	median	(range)	median	(range)	*p*-value
Resources managed					
Items managed (out of 51 trauma resources)	40	(34–51)	47	(35–51)	0.09
Stock keeping					
Stock cards available	35	(33–45)	44	(25–51)	0.52
Stock cards updated	24	(0–45)	31	(0–45)	0.83
Supply chain performance					
Stock-out on day of visit	4	(2–14)	1	(0–5)	0.05
Stock-out within 6 months	9	(3–16)	2	(0–7)	0.07
Number of stock-outs within 6 months	9	(3–57)	2	(0–9)	0.09
Number of days stock-out	171	(51–1268)	8	(0–182)	0.11

### Storage conditions

There was no difference in the overall storage conditions between low and high resource availability hospitals with regard to storage conditions (see Table 4; e.g., organized with regard to first to expire, first out; resource damage, protected from temperature extremes, humidity, water and sunlight; insect and rodent control; security; fire safety; overall *p* = 0.55).

**Table 4:** Trauma care resource storage conditions at low and high resource availability hospitals in Ghana

	Low resource availability hospitals		High resource availability hospitals	
	n	(%)	n	(%)
Products are arranged properly	3	(60)	1	(20)
Products are stored per ‘first to expire, first out’	3	(60)	5	(100)
Cartons are in good condition (e.g., not damaged, wet)	4	(80)	3	(60)
Expired or damaged products are stored separately	5	(100)	5	(100)
Products are protected from direct sunlight	4	(80)	4	(80)
Products are protected from water and humidity	5	(100)	5	(100)
Storage areas are free from insects and rodents	3	(60)	4	(80)
Storage area secured with a lock and key	5	(100)	5	(100)
Products are stored at appropriate temperature	3	(60)	5	(100)
Roof is in good condition to avoid water penetration	4	(80)	5	(100)
Storeroom is in good condition (cleaned)	4	(80)	3	(60)
Current space is sufficient	1	(20)	1	(20)
Fire safety equipment is available and accessible	4	(80)	2	(40)
Products are stored separately from insecticides and chemicals	3	(60)	4	(80)
Products are stacked at least 30 cm away from the walls and other rows	1	(20)	1	(20)
Products are stacked no more than 2.5 m high	0	(0)	3	(60)
Products are stacked at least 10 cm off the floor	1	(20)	2	(40)

Properly was defined as when items are arranged so that identification labels and expiry dates and/or manufacturing dates are visible; FEFO- First to expire, first out; storage area was checked for traces of bats and insects droppings; storage was assessed if it was secured with a lock and key but accessible as and when needed to only authorized personnel

### Stock-outs

Stock-outs of trauma resources were more common in low resource availability hospitals compared to high resource availability hospitals (39 vs 17 facilities with stock-outs in prior 6 months, respectively; *p* = 0.01) (Table 5). Similarly, stock-outs tended to be longer in low resource availability hospitals than in high resource availability hospitals over a six-month period before assessment (total of 180 vs 5 days, respectively; *p* = 0.02). Stock-outs were common even among low-cost, essential resources (e.g., nasal cannulas and oxygen masks, endotracheal tubes, syringes, sutures, sterile gloves).

**Table 5:** Trauma resource stock-outs at low and high resource availability hospitals in Ghana

	Low resource availability hospitals					High resource availability hospitals				
Item	Number of facilities that have experienced stockout in last 6 months	Number of stock out in 6 months		Days of stockout in 6 months		Number of facilities that have experienced stockout in last 6 months	Number of stock out in 6 months		Days of stockout in 6 months	
	*n* = 5	Median	(range)	Median	(range)	*n* = 5	Median	(range)	Median	(range)
Airway (A) resources										
Oral airways	0	0		0		0	0		0	
Nasal airways	1	0	(0–10)	0	(0–180)	0	0		0	
Nasal cannula	2	0	(0–10)	0	(0–180)	0	0		0	
Oxygen masks	2	0	(0–5)	0	(0–180)	0	0		0	
Endotracheal tubes	3	1	(0–1)	10	(0–180)	0	0		0	
Bougie	2	1	(0–1)	14	(0–180)	1	0	(0–1)	0	(0–30)
Endotracheal and tracheostomy ties	1	0	(0–1)	0	(0–24)	0	0		0	
Yankauer tips	2	0	(0–1)	0	(0–175)	0	0		0	
Suction tubing	2	0	(0–1)	0		0	0		0	
Nasogastric tubes	1	0	(0–1)	0	(0–17)	0	0		0	
Cervical collars	2	1	(0–1)	10	(0–53)	1	0	(0–1)	0	(0–30)
Breathing (B) resources										
Chest tubes	0	0		0		1	0	(0–1)	0	(0–12)
Underwater seal containters	0	0		0		0	0		0	
Bag-valve-mask	1	0	(0–1)	0	(0–180)	0	0		0	
Ventilator circuit tubing	0	0		0		0	0		0	
Circulation (C) resources										
Blood pressure cuffs	1	0	(0–1)	0	(0–90)	1	0	(0–2)	0	(0–24)
Spirit (isopropyl alcohol)	1	0	(0–1)	0	(0–14)	1	0	(0–1)	0	(0–90)
Absorbent cotton wool	2	0	(0–7)	0	(0–39)	1	0	(0–1)	0	(0–10)
IV cannulas	0	0		0		0	0		0	
IV fluid administration sets	1	0	(0–1)	0	(0–7)	0	0		0	
Blood administration sets	0	0		0		0	0		0	
Intraosseous needles	0	0		0		0	0		0	
Central venous access sets	1	0	(0–5)	0	(0–180)	0	0		0	
Syringes	3	1	(0–2)	14	(0–21)	0	0		0	
Sharps disposable container	0	0		0		0	0		0	
Cardiac monitor leads	1	3	(0–5)	90	(0–180)	1	0		0	
Electrocardiogram leads	1	1		13		0	0		0	
Electrocardiogram paper	1	1		16		0	0		0	
Defibrillator pads	0	0		0		0	0		0	
Urine dipsticks	0	0		0		1	0	(0–1)	0	(0–5)
Urinary catheters	0	0		0		0	0		0	
Urinary and drainage catch-bags	0	0		0		0	0		0	
Disability (D) and exposure (E) resources										
Splint padding material	0	0		0		0	0		0	
Plaster of Paris	0	0		0		0	0		0	
Gloves (examination)	0	0		0		1	0	(0–1)	0	(0–3)
Face masks	0	0		0		0	0		0	
Eye protection	0	0		0		0	0		0	
Adjunct and other resources										
Xray development supplies	0	0		0		0	0		0	
Ultrasound gel	1	0	(0–1)	0	(0–10)	0	0		0	
Iodine or chlorhexidine	0	0		0		1	0	(0–1)	0	(0–15)
Disposable needles	0	0		0		0	0		0	
Otoscope tips	2	1	(0–1)	13	(0–16)	1	0	(0–1)	0	(0–30)
Glucose test strips	0	0		0		0	0		0	
Finger stick lancets	1	0	(0–1)	0	(0–14)	0	0		0	
Insulin needles	1	0	(0–5)	0	(0–180)	0	0		0	
Surgical resources										
Scalpel blades	0	0	(0–1)	0	(0–14)	0	0		0	
Surgical drains	0	0		0		1	0	(0–1)	0	(0–60)
Sutures	2	0	(0–2)	0	(0–30)	0	0		0	
Sterile gauze	1	0	(0–1)	0	(0–15)	1	0		0	
Sterile bandages	0	0		0		0	0		0	
Gloves (sterile)	0	0		0		4	1	(0–3)	5	(0–15)
	39	10		180		17	1		5	
	*p* = 0.01	0.23		0.22						

### Correlation between resource availability and stock management practices

**Fig. 1:**
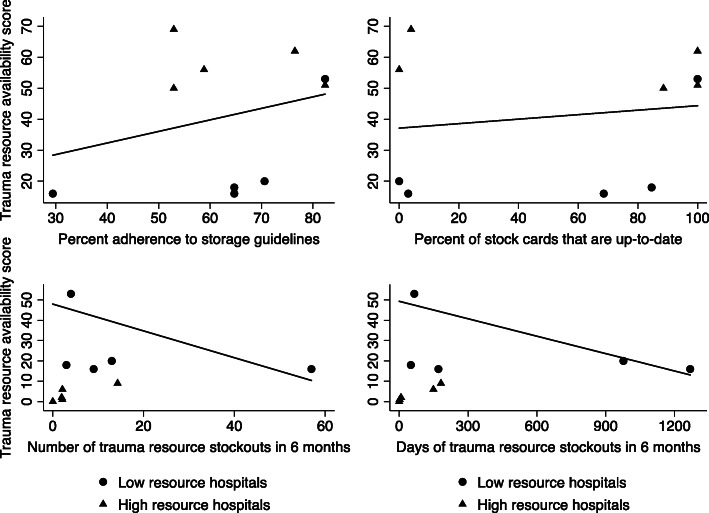
Correlation between multiple aspects of supply chain management structures and processes and trauma resource availability scores at low and high resource hospitals in Ghana

Increased adherence to storage guidelines and higher percentage of up-to-date stock cards were correlated with higher trauma resource availability scores (Fig. 1). Greater numbers of stock-outs and lengths of stock-outs were correlated with lower trauma resource availability scores. However, the variance in trauma resource availability scores was poorly explained by these individual factors or when analyzed in a multivariate regression model (r^2^ = 0.72; *p* value for each covariate between 0.17–0.34).

## Discussion

We aimed to specifically assess trauma care resource-related supply management structures and processes at health facilities in Ghana to inform opportunities to improve the availability of life- and limb-saving services. There were several main findings. First, stock management systems were present in all hospitals; however, high resource availability hospitals more often had frequent inspections, up-to-date stock cards, less frequent stock-outs, and shorter stock-outs. This highlights the importance of active stock management practices. Second, there was generally a low- to moderate-adherence with stock storage guidelines, which places essential resources at risk of damage, waste and stock-outs. Lastly, stock-outs of inexpensive essential trauma care resources were commonly reported, particularly at low resource availability hospitals. In general, organization, standardization, protocol compliance and accountability were identified as broad issues that could improve the availability of specific resources when needed. By highlighting stock management deficiencies and vulnerabilities, we can better inform the planning and organization of trauma care services.

Stock-outs of essential medicines at the hospital level have been widely reported in sub-Saharan Africa and represent a significant public health challenge with a recognized negative impact on morbidity, mortality and disease epidemiology [28]. Although there are a multitude of possible root causes for stock-outs, hospital-level stock management is a common cause of stock-outs and readily addressable with dedicated planning and organization, particularly for services, like trauma care, that have not been prioritized by national health planning activities [8, 17, 29]. In addition to promoting compliance with existing government, USAID, and World Health Organization standards regarding supply chain and stock management best practices, using modeling techniques, training programs, more frequent audits, demand-side incentives, and automated logistic management information systems could markedly improve the availability of trauma resources for injured patients when needed [28, 30]. An intervention study in Mozambique that included fifteen hospitals exposed to standard practice or increased frequency of audits, stock management performance reports and incentives for good performance for family planning resources demonstrated fewer and shorter stock-outs in the intervention group [30]. A recent randomized trial in Zambia examined the effect of three supply chain structures on the frequency and duration of stockouts of essential medicines [31]. The three arms were: Zambia’s existing system, a two-tier system where product is centrally stored as lay away inventory and district stores were used as a cross-docking point without lay away inventory, and a three-tier system with storage of product centrally, at the district stores, and at hospitals. Essential medicine stockouts were markedly less frequent in the two-tier arm compared to Zambia’s existing system. The authors note that even when supply chain system redesign is likely to bring about marked improvements in the availability of essential resources, it often requires stakeholders to navigate a complex political economy within the overall health system and its actors. In the meantime, conducting routine system performance audits and establishing accountability frameworks can reduce stockouts of essential resources [21, 32]. Given that trauma care is planned and organized at the national, regional and/or local levels without an accountability framework, specific attention must be paid to the supply chain and stock management practices for essential trauma resources to ensure their availability when needed.

Compliance with stock management guidelines improves resource availability, reduces waste and promotes appropriate resource use [33, 34]. Further, provider concerns over stock depletion reduce the use of essential resources when needed. A case study of a tertiary hospital in India demonstrated that improving channels of communication between providers, stock keepers, and an automated vendor management system, increasing the frequency of storage audits, and establishing protocols for stock documentation improved stock management performance indicators (e.g., compliance, documentation, stock-outs) [35]. Such quality improvement initiatives are inexpensive and may have a significant impact on resource availability at the hospital-level.

Some of the most common out-of-stock resources were low-cost (e.g., nasal cannulas and oxygen masks, endotracheal tubes, syringes, sutures, sterile gloves). When not due to insufficient funding, stock-outs of low-cost resources are frequently due to a lack of inventory management and procurement processes, which have been widely reported across sub-Saharan Africa [36, 37]. Ideally stockouts are prevented. However, accountability and alert mechanisms can be used to mitigate the risk of future stock-outs and address the current one(s). Establishing hospital-level low-supply alert mechanisms and real-time catalogs of government medical stores may reduce the number and period of stock-outs. *SMS for Life*, a public-private partnership that uses text-messaging to flag low-stocks of anti-malarial drugs, was able to reduce stock-outs from 79 to 26% at health centers in rural Tanzania [38]. Given *SMS for Life* costs less than US$ 80 per facility per year, similar systems for non-drug consumables could be readily implemented in LMIC hospitals and health systems [39].

This study had a number of limitations that should be taken into consideration prior to interpreting the findings. First, the sample size for this study was small. It was our intention to demonstrate the utility of the USAID LIAT for emergency, trauma and surgical resources at the hospital level, was well as identifying specific opportunities to improve the supply chain of hospitals in Ghana more broadly. We specifically selected hospitals that represented all three levels of care (i.e., tertiary, regional, district/first-level hospitals) and extremes of resource availability to better understand the spectrum of supply chain deficiencies and vulnerabilities. Therefore, the findings may not be representative of the entire country. Second, key informants may have over- or under-reported in their responses to USAID LIAT questions. To reduce risk of reporting bias, storerooms and stock management ledgers were systematically reviewed to verify the answers given by stakeholders. Lastly, we did not assess central ordering, procurement, or delivery to hospitals, which can add to the risk, frequency and duration of stock-outs. Although central supply chain management vulnerability and inefficiencies are important, much can be done at the hospital level to improve resource and service availability. Despite these limitations, the findings allow reasonable conclusions to be drawn regarding ways the importance of investigating supply chain management practices and opportunities to strengthen stock management practices at the hospital level in Ghana.

## Conclusions

Good supply chain and stock management practices are correlated with high trauma care resource availability. The findings from this study demonstrate several opportunities to improve stock management practices, particularly at low resource availability hospitals. Governments, health systems and hospitals might consider the following recommendations to ensure that trauma care and other essential resources are available when needed [13, 17, 18, 40]:

- Systematically assess the supply chain and stock management practices to identify opportunities for quality improvement, particularly for resources that lack an accountability framework associated with high priority global health conditions;
- Determine what interventions may improve supply chain and stock management practices based on root cause analyses and assessment of the know-do gap (e.g., incorporate planning exercises, training programs, frequent audits, demand-side incentives, and automated logistic management information systems into hospital stock management programs to improve resource availability);
- Establish hospital-level low-supply alert mechanisms and real-time catalogs of government medical stores to avoid stock-outs of frequently used resources.

## Data Availability

The data collected for this study are available from the corresponding author on reasonable request.

## Abbreviations

PHC: Primary health center
LMIC: Low- and middle-income country (ies)
USAID: United States Agency for International Development
LIAT: Logistics Indicators Assessment Tool

## References

[1] GBD Collaborators (2015). Global, regional, and national incidence, prevalence, and years lived with disability for 301 acute and chronic diseases and injuries in 188 countries, 1990–2013: a systematic analysis for the Global Burden of Disease Study 2013. Lancet (London, England).

[2] GBD Compare Data Visualization [Internet] (2016). IHME, University of Washington.

[3] Wong EG, Gupta S, Deckelbaum DL, Razek T, Kushner AL. Prioritizing injury care: a review of trauma capacity in low and middle-income countries. J Surg Res. 2015;193(1):217–22. 10.1016/j.jss.2014.08.055. Epub 2014 Sep 4. 10.1016/j.jss.2014.08.055 25277355

[4] Mock C, Project ETC, Organization WH, Surgery ISo, Trauma IAftSo, Care SI, et al. Guidelines for essential trauma care: world health organization; 2004.

[5] Mock C. JC, Brundage S, Goosen J, Joshipura M guidelines for trauma quality improvement programmes. Geneva: World Health Organization; 2009.

[6] Mock C, Violence WHO, Prevention I (2010). Strengthening Care for the Injured: success stories and lessons learned from around the world.

[7] USAID | DELIVER PROJECT TO (2009). Logistics System Assessment Tool (LSAT).

[8] Stewart BT, Quansah R, Gyedu A, Ankomah J, Donkor P, Mock C (2015). Strategic assessment of trauma care capacity in Ghana. World J Surg.

[9] USAID | DELIVER PROJECT TO (2008). Logistics Indicators Assessment Tool (LIAT).

[10] Mangan J, Lalwani C, Butcher T. Global logistics and supply chain management. West Sussex: Wiley; 2008.

[11] Stadtler H, Stadtler H, Kilger C, Meyr H (2015). Supply chain management: an overview. Supply Chain Management and Advanced Planning. Springer Texts in Business and Economics.

[12] Ankomah J, Stewart BT, Oppong-Nketia V, Koranteng A, Gyedu A, Quansah R, et al. Strategic assessment of the availability of pediatric trauma care equipment, technology and supplies in Ghana. J Pediatr Surg. 2015;50(11):1922–7. 10.1016/j.jpedsurg.2015.03.047. Epub 2015 Mar 26. 10.1016/j.jpedsurg.2015.03.047 PMC4583787 25841284

[13] Raykar NP, Yorlets RR, Liu C, Goldman R, Greenberg SLM, Kotagal M (2016). The how Project: understanding contextual challenges to global surgical care provision in low-resource settings. BMJ Glob Health.

[14] Gerberg L, Bahirai E, Patykewich L, Cahaelen L (2012). High Impact Practices in Family Planning (HIP). Supply chain management: investing in contraceptive security and strengthening health systems.

[15] Ahmed F, Michelen S, Massoud R, Kaafarani H (2016). Are the SDGs leaving safer surgical systems behind?. Int J Surg.

[16] Reynolds TA, Stewart B, Drewett I, Salerno S, Sawe H, Toroyan T, et al. The impact of trauma Care Systems in low- and Middle-Income Countries. Annu Rev Public Health. 201738:507–32. 10.1146/annurev-publhealth-032315-021412. Epub 2017 Jan 11. 10.1146/annurev-publhealth-032315-021412 28125389

[17] Yadav P (2015). Health product supply chains in developing countries: diagnosis of the root causes of underperformance and an agenda for reform. Health Syst Reform..

[18] Agrawal P, Barton I, Bianco RD, Hovig D, Sarley D, Yadav P (2016). Moving medicine, moving minds: helping developing countries overcome barriers to outsourcing health commodity distribution to boost supply chain performance and strengthen health systems. Glob Health Sci Pract..

[19] Kuupiel D, Tlou B, Bawontuo V, Drain PK, Mashamba-Thompson TP (2019). Poor supply chain management and stock-outs of point-of-care diagnostic tests in upper east Region's primary healthcare clinics, Ghana. PLoS One.

[20] Kuupiel D, Bawontuo V, Drain PK, Gwala N, Mashamba-Thompson TP (2019). Supply chain management and accessibility to point-of-care testing in resource-limited settings: a systematic scoping review. BMC Health Serv Res.

[21] Larson C, Burn R, Minnick-Sakal A, Douglas MO, Kuritsky J (2014). Strategies to reduce risks in ARV supply chains in the developing world. Glob Health Sci Pract.

[22] Stewart BT, Quansah R, Gyedu A, Boakye G, Abantanga F, Ankomah J, et al. Serial assessment of trauma care capacity in Ghana in 2004 and 2014. JAMA Surg. 2016;151(2):164–71. 10.1001/jamasurg.2015.3648. 10.1001/jamasurg.2015.3648 PMC9438847 26502036

[23] Lakerveld AV, Tulder RV (2017). Managing the transition to sustainable supply chain management practices: evidence from Dutch leader firms in sub-Saharan Africa. Rev Soc Econ.

[24] Manso JF, Annan J, Anane SS. Assessment of Logistics Management in Ghana Health Service. Int J Bus Soc Res. 2013;3(8):75–87.

[25] Bossert T, Bowser D, Amenyah J (2014). Ghana: Decentralization and the Health Logistics Systems.

[26] USAID | DELIVER PROJECT TO (2011). Guide to Conducting Supply Chain Assessments Using the LSAT and LIAT.

[27] Bossert TJ, Bowser DM, Amenyah JK (2007). Is decentralization good for logistics systems? Evidence on essential medicine logistics in Ghana and Guatemala. Health Policy Plan.

[28] Leung NH, Chen A, Yadav P, Gallien J (2016). The impact of inventory management on stock-outs of essential drugs in sub-Saharan Africa: secondary analysis of a field experiment in Zambia. PLoS One.

[29] Wagenaar BH, Gimbel S, Hoek R, Pfeiffer J, Michel C, Manuel JL (2014). Stock-outs of essential health products in Mozambique - longitudinal analyses from 2011 to 2013. Trop Med Int Health.

[30] Vermandere H, Galle A, Griffin S, de Melo M, Machaieie L, Van Braeckel D (2017). The impact of facility audits, evaluation reports and incentives on motivation and supply management among family planning service providers: an interventional study in two districts in Maputo Province, Mozambique. BMC Health Serv Res.

[31] Vledder M, Friedman J, Sjoblom M, Brown T, Yadav P (2019). Improving supply chain for essential drugs in low-income countries: results from a large scale randomized experiment in Zambia. Health Syst Reform.

[32] Shieshia M, Noel M, Andersson S, Felling B, Alva S, Agarwal S (2014). Strengthening community health supply chain performance through an integrated approach: using mHealth technology and multilevel teams in Malawi. J Glob Health.

[33] Trap B, Todd CH, Moore H, Laing R (2001). The impact of supervision on stock management and adherence to treatment guidelines: a randomized controlled trial. Health Policy Plan.

[34] Mullany LC, Newton S, Afari-Asiedu S, Adiibokah E, Agyemang CT, Cofie P (2014). Cumulative effects of heat exposure and storage conditions of oxytocin-in-Uniject in rural Ghana: implications for scale up. Glob Health Sci Pract..

[35] Kumar A, Cariappa MP, Marwaha V, Sharma M, Arora M (2016). Improving medical stores management through automation and effective communication. Med J Armed Forces India.

[36] Pasquet A, Messou E, Gabillard D, Minga A, Depoulosky A, Deuffic-Burban S (2010). Impact of drug stock-outs on death and retention to care among HIV-infected patients on combination antiretroviral therapy in Abidjan, Cote d'Ivoire. PLoS One.

[37] Watsierah CA, Ouma C (2014). Access to artemisinin-based combination therapy (ACT) and quinine in malaria holoendemic regions of western Kenya. Malar J.

[38] Barrington J, Wereko-Brobby O, Ward P, Mwafongo W, Kungulwe S (2010). SMS for life: a pilot project to improve anti-malarial drug supply management in rural Tanzania using standard technology. Malar J.

[39] Githinji S, Kigen S, Memusi D, Nyandigisi A, Mbithi AM, Wamari A (2013). Reducing stock-outs of life saving malaria commodities using mobile phone text-messaging: SMS for life study in Kenya. PLoS One.

[40] A Process Guide and Toolkit for Strengthening Public Health Supply Chains through Capacity Development. Geneva, Switzerland: United Nations Children’s Fund (UNICEF) and United States Agency for International Development (USAID), Division US; 2016.

